# Early outcome detection for COVID-19 patients

**DOI:** 10.1038/s41598-021-97990-1

**Published:** 2021-09-16

**Authors:** Alina Sîrbu, Greta Barbieri, Francesco Faita, Paolo Ferragina, Luna Gargani, Lorenzo Ghiadoni, Corrado Priami

**Affiliations:** 1 Department of Computer Science, University of Pisa, Pisa, Italy; 2 Department of Clinical and Experimental Medicine, University of Pisa, Pisa, Italy; 3 Institute of Clinical Physiology, National Research Council, Pisa, Italy

**Keywords:** Risk factors, Data mining, Machine learning, Predictive medicine, Predictive markers

## Abstract

With the outbreak of COVID-19 exerting a strong pressure on hospitals and health facilities, clinical decision support systems based on predictive models can help to effectively improve the management of the pandemic. We present a method for predicting mortality for COVID-19 patients. Starting from a large number of clinical variables, we select six of them with largest predictive power, using a feature selection method based on genetic algorithms and starting from a set of COVID-19 patients from the first wave. The algorithm is designed to reduce the impact of missing values in the set of variables measured, and consider only variables that show good accuracy on validation data. The final predictive model provides accuracy larger than 85% on test data, including a new patient cohort from the second COVID-19 wave, and on patients with imputed missing values. The selected clinical variables are confirmed to be relevant by recent literature on COVID-19.

## Introduction

The Coronavirus Disease 19 (COVID-19) has become a worldwide pandemic, causing by the end of December 2020 over 70 million confirmed cases and over 1.5 million deaths^1^. Hospitals and health facilities are under strong pressure due to limited resources compared to the patient affluence. This is particularly true during the local peaks of the pandemic, when in some regions Intensive Care Units and Emergency Departments (ED) have reached their maximum capacity. Under these conditions, clinical decision support systems based on predictive analytics can help to effectively improve the management of the emergency. In particular, early detection of COVID-19 patients who are likely to develop critical illness and death can aid in delivering suitable care and in optimizing the use of limited resources.

COVID-19 typically manifests as a respiratory pathology, with a wide range of severity and clinical presentation. A considerable fraction of patients show mild symptoms or are asymptomatic. Many patients develop acute respiratory distress syndrome^2^, sometimes followed by a systemic response with several other complications, including thrombotic, cardiac, kidney, gastrointestinal, and neurological effects^3^. Numerous clinical studies, including meta-analyses^4^, characterise the disease on different patient cohorts^4,5^, identify comorbidities and risk factors^2–4,6,7^, and study the effect of different therapy approaches tested around the world^5,8^. The detailed mechanisms of the disease still remain to be uncovered, hence the evaluation of risk at hospitalisation time is difficult to perform, especially in patients with several risk factors. At the same time, while vaccines are currently available, it will take some time before they can be administered to enough individuals to achieve proper population coverage^9,10^. At the same time, virus variants continue to appear, while the best therapy is still to be confirmed^11^.

Predictive models and artificial-intelligence-based technologies can be employed to assist the risk evaluation procedure and have already been employed for various COVID-19 prediction tasks. These include analysis of imaging data, where lung X-Ray or CT scans can be automatically analysed to guide and improve COVID-19 diagnostic and prognosis^12,13^. In particular, a large amount of work has been performed in trying to distinguish between COVID-19 and other pneumonia, or assessing severity, by employing classification with machine learning methods^13^. Diagnostic tools based on clinical data were also developed^14–16^. A different category of methods is using predictive models for prognostic studies based on clinical data, with a large number of models appearing in the last months, e.g.^6,17–23^. Recent reviews^24,25^ show that most of these models are not mature enough to be used for decision making, and require further study.

We present a study that predicts clinical outcomes based on clinical data. The objective is to classify patients into two classes according to the final outcome: discharged or deceased. We start with a cohort of 265 COVID-19 patients hospitalised during the first COVID-19 wave at the Pisa University Hospital, in Italy, including 85 clinical variables measured at the time of admission to the ED. We employ a hybrid filter/wrapper feature selection algorithm to select 6 clinical predictive variables, namely: age, P/F ratio, troponin level, blood urea nitrogen (BUN) level, presence of chronic obstructive pulmonary disease (COPD) and presence of myalgia. These variables are validated with exiting literature, and employed to build predictive models based on logistic regression (LR), decision trees (DT), random forests (RF), naive bayes (NB) and support vector machines (SVM). We perform cross validation on all the patients that have those variables measured (185 patients). We also compare our method with standard filter feature selection. We then validate the selected features on a new dataset with 387 patients hospitalised in the same hospital units during the second COVID-19 wave. Further validation is performed on an extended first wave dataset, where all patients are considered, and missing data is imputed using a nearest neighbours approach. Our selected variables allow for good performance on both the first and second wave datasets: accuracy up to 90% on the first and 86% on the second wave data, and F1-score up to 0.89 on the first and 0.85 on the second wave data. Further interpretability is provided by studying the logistic regression and decision tree classification models and clustering of clinical variables, in order to validate the models from the medical perspective.

## Data and analysis

Our analysis includes two datasets covering patients hospitalised in three different units of the Pisa University Hospital (Emergency Room, Emergency Medicine Department and ICU), during the first and second wave of COVId-19. The data was manually curated, combining both paper and electronic records from the three different units. The dataset contains over 125 clinical variables, some of which are measured several times during hospitalisation. These include: (1) comorbidities, (2) clinical symptoms, (3) physiological variables (blood pressure, respiratory data), (4) blood analysis, (5) current therapies. Available outcomes include final outcomes—dismissal at home or death—and intermediate outcomes—ICU admission, duration of hospitalisation, invasive ventilation and other complications.

The *first wave dataset* covers 313 patients admitted between the 3rd of March 2020 and the 30th of April 2020^26^. We selected the variables measured at the moment of hospitalisation (85 variables) and the patients for which the final outcome is known, discharged or dismissed (265 patients). The rest of the patients were transferred to other units so the final outcome is not available in our data. For the variables measured several times during the hospitalisation period, if the measurement was missing in the first day of hospitalisation, we considered the measurement taken during the second day. This is an approximation but we expect that the values did not change so much from one day to another, and in this way we decreased the number of missing values for these variables. Among the 265 patients, 194 survived and were discharged. The median age across all patients considered is 69, the youngest patient is 18 and the oldest 98. A large part of the patients are male (180 out of 265).

The *second wave dataset* includes 510 patients admitted between the 3rd of September and the 24th of December 2020. Among these, 387 patients were discharged or deceased, while the rest were transferred so the outcome is unknown. Out of 387, 296 survived and were discharged. The median age across the 387 patients considered is 71, the youngest patient is 23 and the oldest 91. Again, a large part of the patients are male (236 vs. 151 female). This dataset was employed for validation of selected clinical variables, hence only patients with those variables measured were extracted (see the “Results” section for details on the number of patients for each clinical variable subset).

All the first wave patients in the analysis were managed with a homogeneous therapeutic regimen, in line with COVID-19 management guideline drawn up by hospital experts^27^.

### Analysis

The analysis has two closely interconnected objectives. The first objective is to select a set of clinical variables that have predictive power when it comes to final clinical outcomes, and can be used for clinical decision making. The selection of clinical variables is guided by an automatic *hybrid filter/wrapper feature selection* method based on genetic algorithms (GA) and logistic regression, that we describe below in "Feature selection for clinical variables based on genetic algorithms" section. An important characteristic of our method is the fact that it is applicable to data with missing values. Standard feature selection methods, and in general machine learning predictive models, require a full data matrix. Therefore before any analysis can be started, the data needs to be cropped to remove missing values, which may include arbitrariness and result in data loss. Our algorithm removes the cropping phase. We compare the variables selected by our method with variables selected by standard feature selection procedures, applied on data after cropping ("Recursive feature elimination" section). The second objective is to build a predictive model that is able to identify critical patients at hospitalisation time. We use five standard classification models, i.e. logistic regression (LR), decision trees (DT), random forests (RF), naive bayes (NB) and support vector machines (SVM), to achieve this objective, and we employ the clinical variables selected at the first step, as described in "Classiffcation models" section. We also discuss interpretability employing logistic regression and decision trees. While feature selection is performed starting from the first wave dataset, predictive performance is tested on both first and second wave data, to validate the features selected.

Further validation of the selected variables and predictive models is performed by missing value imputation with a nearest-neighbour approach described in "Imputation of missing values" section. The imputed dataset is then clustered using hierarchical clustering ("Clustering" section) in order to provide further context to the selected clinical variables.

## Results

### Selection of clinical variables

**Table 1:** Selected clinical variables and their corresponding rankings on the 5 patient subgroups.

Clinical variable	Rank 1	Rank 2	Rank 3	Rank 4	Rank 5
P/F	1	1	1	1	2
Age	2	2	2	2	1
COPD	4	3	3	3	5
Troponin	3	5	9	4	3
BUN	6	4	4	13	6
Myalgia	9	9	6	6	4

**Figure 1:**
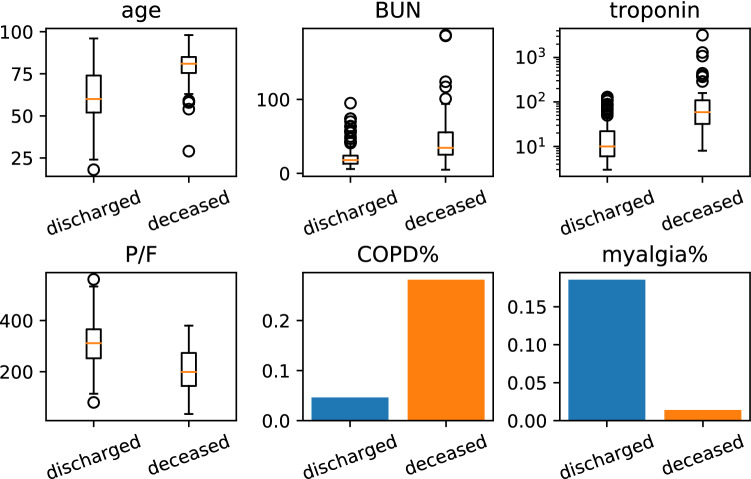
Selected clinical variables. Comparison of values over the two classes of patients.

The genetic algorithm (GA) feature selection method described in "Methods" section produces five different rankings of clinical variables, corresponding to the five patient subgroups from five-fold cross validation (see supplementary material for the rankings and independent validation performance on each subgroup). We consider the top 4 clinical predictive variables from each ranking, thus obtaining the 6 variables listed in Table 1 together with their ranking on the different patient subgroups. We note that the final list contains at least 5 out of the top 6 variables for each patient subgroup. This good overlap among the top variables shows that rankings are robust to changes in patient groups. The supplementary material includes a complete robustness analysis, where we show that the feature selection procedure is also robust to changes in parameter values, giving us confidence that the selected variables are representative of the disease and not of the patient list or search settings. Among the variables selected, age and the P/F ratio are the top ranked, followed by COPD, Troponin and BUN. The presence of myalgia is in the top 4 in only one patient subgroup, and ranks 6 in two other groups. We do however include this variable as well, and evaluate the final performance.

Figure 1 shows the distribution of the selected variables over the two clinical outcomes. The six predictive variables have a strong support in the medical literature on COVID-19. Higher age is one of the most cited risk factors^2,4,6,18,22,28^, and our algorithm correctly identifies it. The P/F ratio is an important variable with lower values indicating worse respiratory function. Given that the respiratory system is one of the main systems affected by the progression of COVID-19, P/F has been used in clinical decision making for external ventilation and oxygenation of patients^29,30^. Another respiratory variable we select is COPD, which flags the patients having chronic lung disease. Again, this co-morbidity has been previously found to increase the risk of a poor outcome in COVID-19 patients^31–33^.

Another important aspect related to multi-organ failure and co-morbidities that our selection algorithm is able to recognise is the link between COVID-19 and the cardiovascular system, through the selection of the troponin clinical variable. The correlation between mortality and elevated troponin values could highlight acute organ damage, already suggested in the literature with acute myocarditis^2,3,34–37^. On the other hand, the pre-existence of cardiovascular pathology could be responsible for a transient increase of this value, as a result of a greater cardiac susceptibility in some COVID-19 patients. In our dataset, troponin is a clinical variable that is missing in 57 out of the 265 patients, and it will not be selected under some settings of the algorithm (see Methods and Supplementary material for more details). Even so, the variable is at the top of the rankings, underlining its role in the disease.

Another clinical variable selected by our automatic method is BUN. Elevated BUN levels can be linked to a pre-existing chronic kidney disease but also to a situation of hypovolemia and hypoperfusion determined by the septic state. It could therefore be interpreted as a marker of infection severity and related mechanisms. The link between BUN and mortality has been observed in COVID-19 patients also in other studies^3,6,17,18,38,39^.

The last variable selected by our algorithm is myalgia. Presence of myalgia appears to be an advantage in the case of COVID-19, with lower incidence among the deceased patients compared to those discharged (see also^4^). The identification of “protective” factors from negative outcomes of COVID infection, such as myalgia, could suggest that the disease could develop against different target organs, depending on the case. When the target organ is the lung, the disease is expressed with the most fearful respiratory manifestation. Instead, in other cases, the disease could refer to other districts, manifesting itself in a mild form.

The existing predictive models also select clinical variables with high prediction power^17,18,22,25^, and they have some overlap with our variables. In general a maximum of two variables are common with our analysis, and they are typically accompanied by several others. Our list is shorter than most predictive analyses published to date, which provides an advantage both in terms of cost and real applicability in the hospital setting. Predictive power varies among other studies, and our accuracy values are in line with the literature.

To compare our method with standard feature selection procedures, we performed recursive feature elimination (RFE, see "Recursive feature elimination" section) using decision trees and logistic regression (DT-RFE and LR-RFE). Table 2 shows the features obtained using 20-fold cross validation, along with the frequency they were selected with. As previously discussed, performing standard feature selection with missing data is not straightforward, as one has to crop the data to remove missing values before starting the analysis. Here we adopt a method where we first select the variables with patient coverage above a threshold, and then we remove patients with missing values (see "Methods" and the supplementary material for details). We report selected features when employing two different coverage thresholds: 90% and 75% patient coverage, resulting in 171 and 74 patients, respectively. For the 90% threshold, we observe that the top two features in both cases are common to those selected by our algorithm, i.e. P/F and age. COPD is selected by the logistic regression model. However the other features are different from the six selected by our method. In particular, troponin and BUN are not selected, because they do not meet the 90% patient coverage threshold. When decreasing the threshold, BUN and troponin can be selected, however they appear only in the DT-RFE method. In the following we will evaluate the predictive performance of these four feature sets and compare with our genetic algorithm. However, it is already obvious that our method provides an advantage in that it removes the arbitrary initial selection of features, and allows for inclusion of important features even with lower coverage, not ignoring at the same time the important features with wide coverage.

**Table 2:** Features selected by recursive feature optimisation (RFE) employing logistic regression (LR) and decision trees (DT).

Coverage threshold	DT-RFE features	LR-RFE features
90% (171 patients, 59 variables)	Age(14), creatinine(14), platelets(18), lymphocytes(19), P/F(20)	Anticoagulants(12), diarrhea(14), chronic liver disease(15), asthma(17), age(20), COPD(20), P/F(20)
75% (74 patients, 68 variables)	Troponin(11), sodium(14), glucose(15), creatinine(19), BUN(20), PCT(20), P/F(20)	PCT(13), immunosuppressives (14), diarrhea(16), asthma(17), age(19), COPD(20), fever(20)

We show two different experiments, with different initial selection of starting clinical variables (coverage of 90% and 75% of patients). In parentheses we show the frequency with which the feature was selected, out of 20 validation runs (20-fold cross validation). A frequency of 20 means features were selected in all validation runs. We only include features selected in over 50% of the runs.

### Prediction accuracy

We use five predictive models to classify patients into discharged or deceased by using the clinical variables discussed in the previous section. In particular, we compare the six variables selected by our GA method with the four variable subsets selected by RFE with different parameter settings, i.e. the variables from Table 2. We fist study the performance on the first wave dataset, the one also employed for feature selection. Table 3 shows Accuracy and F1 score for each method. We note that four out of five models provide classification accuracy over 85%, with best results by LR (Accuracy 0.903, F1-score 0.899) followed by RF, DT and SVM. On these models, our GA algorithm provides superior performance compared to the variables selected by standard methods. For the variables selected among those with 90% coverage, the lower performance is probably attributable to the fact that some important features like troponin and BUN are missing due to the threshold imposed. For the 75% coverage, the RFE procedure is not able to select general features, and this is probably due to the reduced number of patients after initial data cropping (only 75 patients with 68 clinical variables measured). Our method does not crop the data, therefore is able to select a more general set of features.

The performance presented in Table 3 is also superior to simply applying the predictive models on all first wave data, without any feature selection. Similar to feature selection, predictive models require to first crop the data, and we explore various coverage thresholds, all of which provide lower predictive performance. Due to the complexity of the procedure, we discuss full methodological details and results in the supplementary material, "Results" section. All in all, the results demonstrate the two-fold utility of feature selection: reduced number of variables to be monitored and increased predictive performance.

**Table 3:** Classification with leave-one-out cross validation on the first wave dataset. The bold font identifies the models with best performance, for each of the different rows (i.e. for each predictive model type).

	F1 score					Accuracy				
		Coverage 90%		Coverage 75%			Coverage 90%		Coverage 75%	
Model	*GA*	DT-RFE	LR-RFE	DT-RFE	LR-RFE	*GA*	DT-RFE	LR-RFE	DT-RFE	LR-RFE
LR	**0.899**	0.824	0.865	0.809	0.806	**0.903**	0.829	0.870	0.820	0.810
DT	**0.863**	0.766	0.808	0.830	0.734	**0.870**	0.785	0.819	0.832	0.731
RF	**0.874**	0.818	0.808	0.846	0.776	**0.876**	0.825	0.815	0.851	0.778
NB	0.778	0.800	**0.836**	0.775	0.803	0.762	0.801	**0.839**	0.789	0.806
SVM	**0.840**	0.640	0.771	0.824	0.718	**0.859**	0.748	0.799	0.845	0.736
Support	185	246	254	161	216	185	246	254	161	216

Each row corresponds to a different model type. We compare models trained on variables selected by our feature selection method (GA) with those selected by the two recursive feature elimination algorithms with the two different coverage thresholds (DT-RFE, LR-RFE).

Selecting clinical variables based on a limited number of patients always runs the risk of overfitting the cohort analysed^40^. We thus test the methods on the same set of features on the second wave dataset, including patients not used in the features selection phase. Table 4 shows predictive performance on these data, using leave one out cross validation. We note that the only model that maintains accuracy above 85%, and close to that of the first wave data, is the decision tree with our six selected features. The other models have lower performance, maintaining accuracy values above 75%, and a wide gap between the first and second wave. On one hand this result indicates that the features we selected are superior to the RFE method, and in particular the importance of troponin and BUN in the analysis. The importance of the two features is also confirmed by the fact that among the RFE methods the set of features selected by DT-RFE are the best (they are the only one to include troponin and BUN). One observation to be made is that with the inclusion of troponin and BUN the number of patients that have valid data decreases a lot (See Support values in Table 4). This was also true, albeit with a different ration, on the first wave data. Hence we believe that these variables should be monitored better for COVID-19 patients, to allow for improved prediction. Regarding the various predictive models, the superior generalisation ability when employing decision trees indicates the robustness of this simple model, which is probably partly due to the discrete nature of the classification procedure.

**Table 4:** Classification with leave-one-out cross validation on the second wave dataset (validation).

	F1 score					Accuracy				
		Coverage 90%		Coverage 75%			Coverage 90%		Coverage 75%	
Model	*GA*	DT-RFE	LR-RFE	DT-RFE	LR-RFE	*GA*	DT-RFE	LR-RFE	DT-RFE	LR-RFE
LR	0.764	**0.787**	0.744	0.773	0.728	0.778	**0.807**	0.775	0.795	0.772
DT	**0.853**	0.719	0.701	0.790	0.783	**0.861**	0.730	0.718	0.808	0.789
RF	**0.811**	0.725	0.700	**0.811**	0.744	0.819	0.750	0.711	**0.821**	0.764
NB	0.626	0.736	0.712	**0.773**	0.710	0.611	0.723	0.695	**0.782**	0.758
SVM	0.765	0.652	0.659	**0.777**	0.658	0.806	0.757	0.762	**0.808**	0.761
Support	72	296	298	78	360	72	296	298	78	360

Each row corresponds to a different model type. We compare models trained on variables selected by our feature selection method (GA) with those selected by the two recursive feature elimination algorithms with the two different coverage thresholds (DT-RFE, LR-RFE). The bold font identifies the models with best performance, for each of the different rows (i.e. for each predictive model type).

### Imputation of missing values

**Figure 2:**
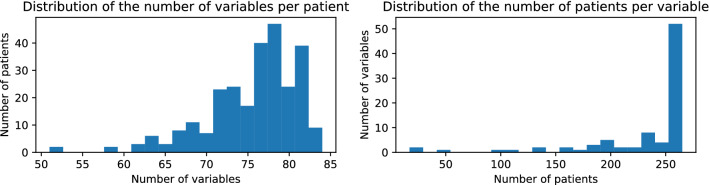
Clinical data and missing values. The plots show the frequency histogram for the distribution of the number of clinical variables available per patient and patients per clinical variable.

**Figure 3:**
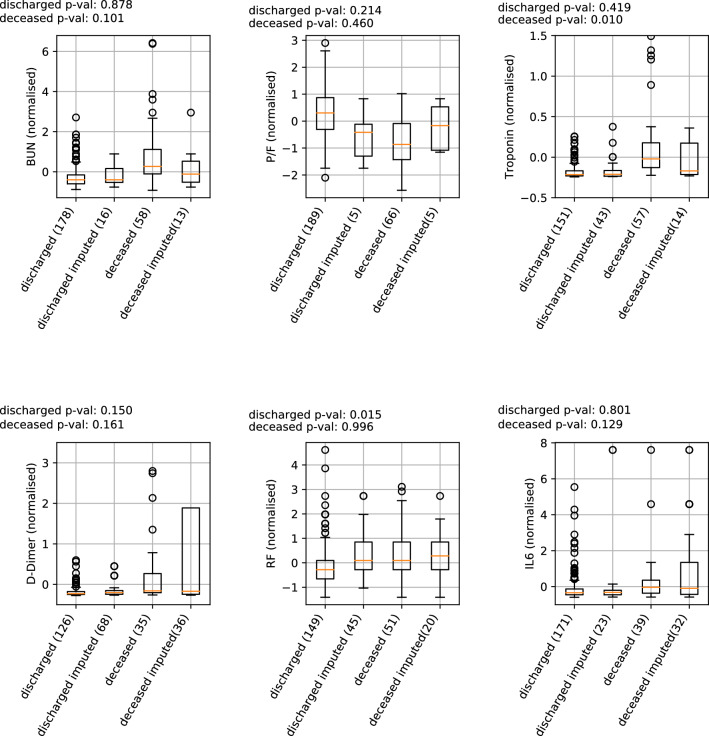
Distribution of a selection of measured and imputed clinical variables for discharged and deceased patients. The plots compare the values measured at hospitalisation with those imputed during our analysis. The *p* values correspond to two-sample Kolmogorov–Smirnov tests, comparing *discharged* versus *discharged imputed* and *deceased* versus *deceased imputed*. The labels on the x-axis show in parentheses the number of patients in each group.

For the first wave dataset, many of the clinical variables are missing in some patients, as shown in Fig. 2. Only 38 variables, out of a total of 85, are available for all patients, making our analysis very difficult. Missing value imputation could help to employ the entire cohort in the analysis, however it will necessarily include some noise. Hence we use the imputation of missing data to test the validity of the selected variables, and not for their initial selection. We impute missing values based on a nearest neighbours approach (see Methods for details). We compare the distribution of variables between the real and imputed patients, to test the level of noise introduced by the imputation. Figure 3 shows boxplots of the distributions for six variables, three of which are among those that we use for prediction: BUN, P/F and troponin. In most cases we observe that there are no significant differences between the real and imputed distributions, as measured also by a two-sample Kolmogorov-Smirnov test. At the same time, differences among the two classes are typically maintained. For the Troponin variable the difference between real and imputed values on the deceased patients is significant at 95% level, however the difference has little effect on the prediction performance, as we will see below.

The imputation of missing values allows us to test the performance of the predictive models using our variables on all first wave patients. Table 5 shows predictive performance on the imputed dataset with all 265 patients. Classification performance remains high for most models. The LR and RF models show a slight decrease in accuracy, compared to the restricted dataset (Table 3, while the NB and SVM slow a slight increase. The decision tree losses more in accuracy. Overall, the best result is obtained with LR, with F1-score and accuracy over 0.87. This analysis provides an additional independent validation of the selected clinical variables and an indication of classification performance on new patients. Furthermore, the results indicate that missing value imputation may be useful in applying this type of analysis on additional patients, which is important in a context where the medical system is under pressure.

**Table 5:** Classification with leave-one-out cross validation on the first wave dataset, with the top six clinical variables selected by our GA algorithm, after imputation of missing values (265 patients).

Model	F1-score	Accuracy
LR	0.872	0.875
DT	0.796	0.804
RF	0.848	0.853
NB	0.819	0.826
SVM	0.854	0.860

Each row corresponds to a different model type.

### Interpretability

**Table 6:** Logistic regression coefficients after leave-one-out cross validation with the clinical variables selected by the GA on the first wave dataset (Table 3, F1-score 0.899, Accuracy 0.903).

Clinical variable	P/F	Age	COPD	Troponin	BUN	Myalgia
LR Coefficient	$-0.01$	0.06	1.33	0.02	0.03	$-1.64$

**Figure 4:**
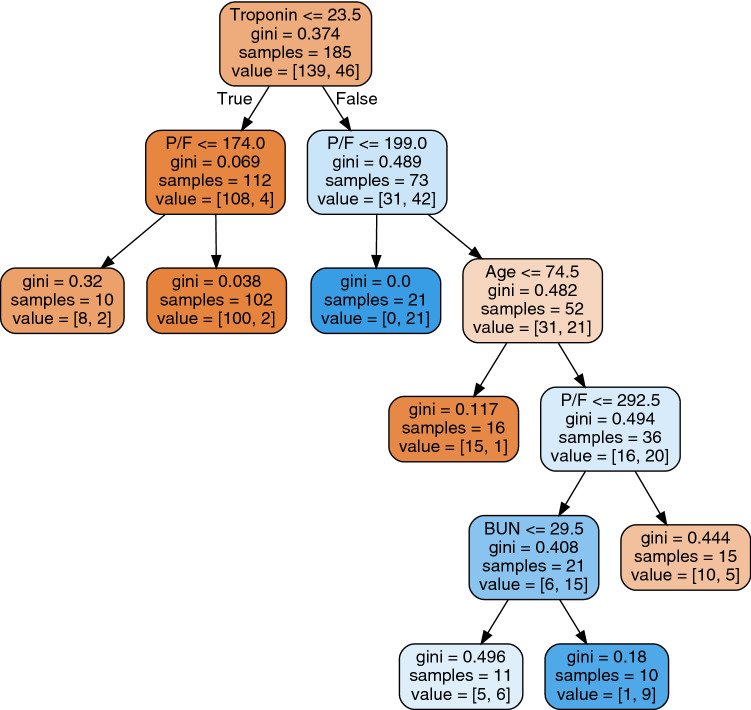
Decision tree trained on all 185 patients that have no missing values among the 6 selected clinical variables. Each node of the tree contains a condition on a clinical variable. We also include the number of patients, and the way they are divided into the two classes (discharged/deceased). The colour of the node shows whether patients are in the discharged class (orange) or deceased class (blue).

Among the five predictive models employed in teh analysis, two are interpretable: the LR and DT models. The LR model is a standard practice in the medical domain, and can be interpreted by studying the regression coefficients. Table 6 shows the coefficients that indicate that higher age, troponin and BUN levels, and history of COPD , result in higher mortality. For P/F and myalgia, the relation is inverse: higher P/F and presence of myalgia result in lower mortality. Our results are in agreement with the scientific medical literature (see "Selection of clinical variables" section for details) supporting the viability of our model for predicting clinical outcomes.

Figure 4 shows a decision tree model, trained on all 185 patients that have the 6 variables selected by our GA procedure. The tree obtains an F1-score of 0.943 and an accuracy of 0.913 on training data. The decision tree provides a visual description of our training dataset, based on the clinical variables, as follows. Our full dataset of 139 discharged patients and 46 deceased patients is interrogated for the troponin levels. If troponin is below 23.5, then we move left in the tree, otherwise we move right. We can observe that 112 patients have low troponin levels and 108 of these survive. On the right side, however, only 31 out of 73 survived. The second level of the tree investigates P/F levels. The limits chosen by the algorithm in the two nodes left and right from the root are close to one another, and close to the level under which the acute respiratory distress syndrome is considered moderate or severe (200). We observe that, among patients with low troponin, a P/F under 174 raises the probability of a negative outcome to 20% in our data (2 out of 10 patients). For large troponin levels (node to the right of the root), all patients with P/F under 199 will not survive, while patients with higher P/F levels have a mixed outcome. This shows exactly how the first two variables work together to decide the outcome. The third level of the tree deals with age (rightmost node), that allows us to divide the 52 patients with high troponin and high P/F into two groups. Patients under 75 survived, while the older ones show again a mixed pattern, that can be further refined using again the P/F and BUN variables.

The decision tree is also a tool to easily classify a new patient, by following the path in the tree based on the values of the clinical variables. It is possible to compute a probability of a negative outcome by looking at the composition of the training data for each node. The tree can be used even if not all clinical variables are available, albeit with lower resolution. For instance, if one has only troponin and P/F available, the analysis can stop at the second level of the tree.

**Table 7:** Clustering of clinical variables.

Cluster	Clinical variables
1	Fatigue, shortness of breath, Haemoglobin, INR, bilirubin, pH,
2	Body temperature, nasal congestion, 24h urinary output, CD4CD8, sodium, pCO2, GM CFS,
3	Fever, respiratory rate, heart rate, ferritin, LDH, PCR, PCT,
4	COPD, cerebrovascular disease, hematological malignancy, BMI,
5	Cancer, chronic liver disease, liver cirrhosis, other pathogens,
6	Sex, smoking, rash,
7	From LTCF, neurological disease, dementia, confusion on admission,
8	Neutrophils, WBC, platelets,
9	Hemoptysis, pleuritic chest pain, haematocrit,
10	Dialysis, chronic kidney disease, creatinine,
11	Diabetes mellitus, hypercholesterolemia, glucose,
12	Conjunctival congestion, pO2,
13	BUN, potassium, CPK, troponin, lactate,
14	Systolic BP, diastolic BP,
15	Autoimmune disease, immunosuppressives ,
16	IL1b,
17	D-Dimer,
18	GM-CSF, MCP, MCP>720,
19	Cough, sore throat, sputum,
20	Symptoms days, BAL,
21	AST/GOT, ALT/GPT,
22	HCO3,
23	IL6, TNFalfa,
24	Age, cardiovascular disease, anticoagulants, IL10,
25	Arterial hypertension, RAAS blockers,
26	Asthma, lymphocytes, P/F,
27	Headache, nausea/vomiting, diarrhea, myalgia,

To better contextualise the selected clinical variables, we perform a clustering of the variables based on the entire cohort of 265 patients after missing value imputation. Table 7 shows the composition of the clusters, with the total number of clusters set to 27. The clusters obtained are generally small and specific to certain diseases or measurement technologies. For instance cluster 10 groups together variables related to kidney diseases, cluster 14 is formed by the two measures for arterial pressure, and cluster 11 includes variables related to cardiovascular risk factors. Some clusters are more heterogeneous and group together variables from different organs/tests. The six variables that we selected for prediction come from five clusters. Troponin and BUN are in cluster 13, a heterogeneous cluster that links kidney, heart and lung disease. COPD is part of cluster 4 and seems to be related in our cohort with vascular conditions. Myalgia is part of cluster 27 which includes other non-respiratory symptoms, such as headache and nausea, indicating again that these other symptoms could be correlated in COVID patients. P/F is in the same cluster with asthma, and lymphocyte levels (cluster 26). Age is part of cluster 24, together with variables related to cardiovascular disease. The heterogeneity of some of the clusters in which the selected variables fall my indicate that they are a proxy for other variables as well.

## Discussion

The analysis presented here identifies six clinical variables that allow us to predict, at the time of hospitalisation, what will be the final clinical outcome: patient discharged or deceased. The identification is based on a feature selection algorithm that uses independent validation to build a ranking of clinical variables.

Our method is designed to overcome several challenges in developing such models. First, the analysis requires the availability of structured clinical data, typically coming from electronic health records. During emergency times, the usage of non-electronic records has prevailed because of the pressure on the health system. Therefore our data needed manual curation at the beginning of the study.

A second challenge is that the set of measured clinical variables differs strongly from patient to patient. This could be due to different approaches of the medical personnel involved, cost restrictions, patient needs and status. A critical patient admitted directly in the intensive care unit (ICU) undergoes a different set of tests compared to a stable patient with acute symptoms. In this context, the identification of predictive variables of clinical outcomes is mandatory to ensure that they are measured for all new patients. Restricting the analysis to patients for whom all variables have been measured would make their set very limited, as would selecting variables that cover a certain fraction of patients, as we show in Table 2 and Supplementary material. Furthermore, the initial cropping of the dataset introduces arbitrariness in the procedure. Thus the analysis needs to adapt to a very sparse matrix, and it requires strong and repeated interactions with medical experts so that relevant variables are not left out, and they are properly “explained”. One typical method of variable selection is to rank all variables based on some criterion, and select the most relevant ones for prediction. A possible ranking can come from univariate or multivariate statistical tests^20^, or from predictive models themselves, as is the case for recursive feature elimination (RFE)^41^. In this work we use a hybrid filter/wrapper feature selection method based on Genetic Algorithms, which dynamically adjusts the patient cohort based on the clinical variables selected. The final result is a ranking of clinical variables based on their predictive power. The resulting ranking is very robust to changes in parameters of the algorithm or in the subset of patients included in the selection phase. The Supplementary Material includes a discussion of robustness, with the full variable ranking for five different patient subsets, where we observe great similarity at the top of the rankings. This gives us confidence that, even if the number of patients in our data is not large, the selected variables are generic and do not simply describe the cohort we are using. The relevance of the variables we select is reinforced by the clinical value they carry, and the amount of literature supporting them.

The third challenge is related to the validation of the AI predictive model: the model must generalise for *variable selection* and for *prediction*, and must report a realistic error rate. To ensure this, the ranking of clinical variables resulting from our feature selection method is based on the performance on independent validation cohorts. The selected predictive variables are validated using cross validation on all the patients from the first wave dataset that have those variables measured (185 patients), and also on a second patient cohort from the second COVID-19 wave (72 patients). Further validation is performed on an extended first wave dataset, where all 265 patients are considered, and missing data is imputed using a nearest neighbours approach. The good performance in all validation settings support again the use of these six variables for decision making. We also demonstrate the superiority of this variable set compared to RFE.

A final challenge is model interpretability: the decision made by the AI method needs to be transparent. This enables both understanding of the final response and validation of the model by using medical knowledge. Examples of interpretable models for COVID-19 patient classification are^20,23^. Here, we use two classification models: logistic regression and decision trees, both of which are so called ’white-box’ models. Logistic regression provides interpretability through the regression coefficients, while the decision tree provides a set of rules used to decide the outcome. The indications from both our models are valid from the medical perspective.

We would like to underline the role of two relevant variables: troponin and BUN. They integrate information on both *pre-existing* cardiac and renal conditions, and organ damage that may occur *during* the infection with COVID-19. Therefore, the use of troponin and BUN is stronger than simply looking at pre-existing comorbidities.

The study presented here concentrated on the prediction of death. A related question could be whether we can distinguish mild from severe disease, where severe disease includes cases where the outcome is either death or ICU admission. Mild cases instead are those who are discharged without any ICU admission. This prediction could be applied, for instance, to select patients for clinical trials. The method we presented can be immediately applied to this related problem, by simply changing the outcome to be predicted. To showcase this, we have retrained the predictive model on this new problem, employing the same six features optimised for predicting death on the first wave dataset. Table 8 shows the predictive performance in this case. We note that, even with the same features, we can predict very well the severe disease cases, with an F1 score superior to that of the death prediction problem, and an accuracy over 90%. This performance could be possibly improved further by optimising the variable selection for this problem as well.

**Table 8:** Classification with leave-one-out cross validation on the problem of predicting severe disease (ICU admission or death), on the first wave dataset, with previously selected clinical variables.

Model	F1-score	Accuracy
LR	0.931	0.930
DT	0.936	0.935
RF	0.931	0.930
NB	0.931	0.930
SVM	0.968	0.968

Each row corresponds to a different model type.

Even though we have made all efforts to follow suitable external validation practices, both during the selection of clinical variables and after, using data from two different COVID-19 waves, the fact that our study is based on data from one hospital only is still a limitation. To demonstrate the validity of our model in changing settings, which is an important aspect before including it in clinical decision making^24,42^, we plan to expand the analysis on larger datasets and cohorts coming from different locations, as soon as they become available. Given that unfortunately we are now facing subsequent waves of the pandemic, we also plan to continue to apply our predictions on data from new patients from the same hospital. The variables we underlined, and the predictive models, could be used all around the world to aid clinical decision making in a time of emergency as the one we are living in.

## Methods

### Ethics statement

This study was performed according to the principles stated in the Declaration of Helsinki and it conforms to standards currently applied in our country. The patient’s informed consent was obtained in line with security protocols in place in the hospital during the emergency. The use of the data was approved by the Comitato Etico Area Vasta Nord Ovest (Internal Review Board—IRB number 230320).

### Data availability

Due to privacy reasons the clinical data cannot be made public without restrictions. However, access can be granted to interested researchers upon motivated request to the authors of this paper, and upon subsequent approval by the interested bodies.

### Analysis

This section describes the various tools employed to achieve the various objectives of our study: selecting the clinical variables relevant for prediction, building and validating the actual predictive models, missing value imputation and clustering of clinical variables. The entire analysis was performed in python, using the library scikit-learn^43^ for the prediction and clustering tasks.

#### Feature selection for clinical variables based on genetic algorithms

**Figure 5:**
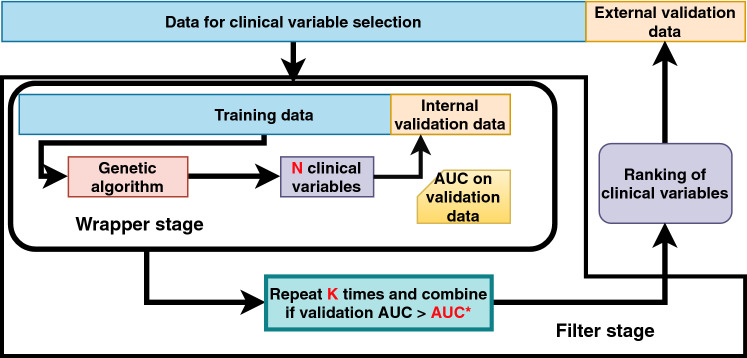
GA feature selection methodology. The result is a ranking of clinical variables, which is then validated on an independent set of patients.

We present a new hybrid filter/wrapper feature selection method based on genetic algorithms that generates a ranking of the predictive power of variables starting from the measured clinical variables. The ranking is then validated on an external dataset, and the top variables are selected to proceed with the prediction. We repeated the entire analysis five times, with five different patient cohorts, extracted from the first wave dataset, for variable selection and external validation, using a 5-fold cross-validation method. This resulted in five different rankings with large similarity especially at the top of the ranking (see Supplementary Material for the rankings and validation performance of each). For the final selection of clinical variables, we opted for the top 3 variables from each ranking. This resulted in the 6 different clinical variables listed in Table 1.

Figure 5 shows the general structure of the method, which consists of a wrapper stage included within a filter stage. At the wrapper stage, a genetic algorithm is employed to generate a set of clinical variables of fixed size, *N*, that classifies well a training dataset. The *N* clinical variables are then tested on an independent validation dataset, using logistic regression, and resulting in a validation AUC value. This validation is denoted as *internal validation*. The procedure is repeated *K* times, changing every time the training and validation subsets, while maintaining their relative size (10% of patients for validation). This resulted in several sets of clinical variables and their corresponding validation AUC. Among these, only those that have good AUC values, over a predefined threshold $\hbox {AUC}^*$, are maintained. They are then combined into the final ranking, by simply counting how many times each clinical variable appears. The ranking is then used at the filter stage of our methodology: logistic regression models with an increasing number of clinical variables from the top of the ranking are built, and independently validated on *external validation* data.

*Genetic algorithm details.* Genetic algorithms are a family of population-based algorithms where solutions to an optimisation problem are generated using concepts from Darwinian evolution^44^. An initial (random) population of solutions evolves in discrete time steps (generations) using two operators: mutation and crossover. Selection is applied at each generation so that the solutions with the best fitness are admitted to the next generation. After several generations, the population is able to explore promising areas of the search space and provide good local solutions. Genetic algorithms have been previously used for wrapper feature selection and for metaparameter optimisation in predictive models, including biomedical applications, due to their efficiency in exploring the search space in few iteration, reducing thus the number of models to be trained (e.g. the recent works of^45–48^). However, to our knowledge, a filter/wrapper hybrid similar to ours does not exist.

Our objective is to select a subset of *N* clinical variables that enables classification of patients into the deceased/discharged classes. Our algorithm generates initial random subsets of a fixed size *N*. The solutions are evaluated by training and testing a logistic regression classifier using leave-one-out cross validation. The fitness of the solutions is given by the AUC values (area under the ROC curve). Solutions are mutated at each generation by replacing one of the clinical variables with a different one. Crossover between two solutions is implemented by splitting the solutions into two parts of randomly generated sizes and swapping them among the parents. The new solutions thus obtained are then assigned a fitness value and added to the population. At each generation, selection is performed by keeping only the solutions with the largest fitness, i.e. largest AUC values.

Fitness evaluation embeds a flexible mechanism that adapts to data with missing values. Typically , when missing values are present, the data is cropped at the start of the analysis by removing both data points and features (patients and clinical variables in our case). However, this has the disadvantage that the size of the data is reduced, and the removal of variables is arbitrary. Here, we perform the cropping only when calculating the fitness for each solution representing a subset of variables, to maximise the number of patients that can be considered. This procedure has the risk that, when the set of variables includes many missing values, the number of patients is very reduced. We control this by a parameter of the genetic algorithm, $p^*$, such that if a set of variables does not contain at least $p^*$ patients, then that set of variables is assigned a fitness of 0. This allows the genetic algorithm to select sets of variables that have at least $p^*$ patients.

*Experimental setup.* The feature selection method has several parameters: the *N* clinical variables that the genetic algorithm looks for; the *K* times the internal wrapper stage is performed; the minimum number $p^*$ of patients for which a set of variables is available; the threshold $\hbox {AUC}^*$ under which solutions are discarded. We performed $K=100$ different optimisation runs, for $N\in {5,10}$ and $p^* \in {50,75,100,125,15,175}$. The final ranking was obtained from all the runs combined, using an AUC threshold $\hbox {AUC}^*=0.85$. The supplementary material includes a discussion on the robustness of the ranking of clinical variables to varying these parameters, and we show that there is large similarity between rankings obtained with different values. The only parameter that has a visible effect is $p^*$. This forces the algorithm to remove the clinical variables that have many missing values. Consequently, variables like troponin or BUN appear only when $p^*$ is small, however they are frequent enough to be selected in the final ranking anyway. For these reasons we decided to combine all the optimisation runs into one final ranking.

*Discussion.* Our hybrid feature/wrapper feature selection method has two important characteristics. First, it is resilient to missing values in the dataset, by embedding a mechanism that adjusts the training data size based on missing values. This removes the need to crop the dataset at the start of the analysis, to remove missing values.

Second, the selection of variables is based on two different independent validation procedures, ensuring that the selected variables are general enough. The first validation procedure is internal, and applies to the solutions of the genetic algorithm, that are filtered based on test AUC. The second validation procedure is external, and applies to the final ranking of clinical variables. The generality of our selected variables is also supported by the results obtained from different patient cohorts. The five rankings from different patient subgroups are very similar at the top of the ranking (see Supplementary Material). In fact, by selecting the top 3 variables of each ranking, we cover 5 variables out of the top 6 of all rankings. Additionally, the performance on independent external validation is very good for all five rankings. When considering the top 6 variables from each ranking, independent validation accuracy is perfectly inline with results presented in the previous section with the final set of 6 variables. We also see good agreement for new validation data from the second COVID-19 wave, for decision trees and random forest models.

#### Recursive feature elimination

We compare the new approach introduced above with a standard method to perform feature selection: recursive feature elimination (RFE)^41^. This method ranks the clinical variables by a criterion, removes the least important variables and then reranks, repeating the procedure until a certain number of variables is obtained. Here we employ this method to obtain 10 clinical features, and we use two different ways to rank features: based on logistic regression (LR) and on decision tree (DT) feature importance values. We perform 20-fold cross validation, and combine the resulting sets of clinical variables, selecting those that appear at lest 10 times in the final RFE solutions.

An important step in the analysis is defining the initial dataset. As mentioned several time, our data suffers from missing values. Hence we need to select a set of variables and patients to be considered as a starting point. We adopt an approach where we first eliminate variables that have many missing values, i.e. we set a *patient coverage threshold* under which variables are removed. Then, with the remaining variables, we eliminate all patients that have at least one missing values. Table 1 in the Supplementary material shows the number of patients and variables for different coverage thresholds, for the analysis here we select two thresholds: 90% and 75%, resulting in 171 and 74 patients, respectively. Hence we perform RFE with DT and LR feature importances, on two different datasets, resulting in four subsets of features, discussed above and included in Table 2.

#### Classification models

Our feature selection method uses internally a logistic regression classifier^49^, both for the wrapper stage and the external validation of the five different rankings produced. During the feature selection phase clinical variables are standardised (scaled to have mean 0 and standard deviation 1) before being fed to the logistic regression model.

The logistic regression model was selected through a preliminary comparison of various predictive models, including Decision Trees^50^, Random Forests^51^, Support Vector Machines^52^ and Naive Bayes^50,52^ classification. The supplementary material shows the predictive performance before feature selection, with different initial cropping of the dataset to remove missing values. Most of the times, logistic regression outperformed the other models, while it was sometimes outperformed by the RF model. However, RF requires a large time for training. Given the large number of models to be trained during the filter/wrapper feature selection phase, we decided to select logistic regression for this task.

Once clinical variables were selected, prediction was studied for five different models: Logistic Regression, Decision Trees, Random Forests, Support Vector Machines and Naive Bayes. These are classical machine learning models, and we used the scikit-learn Python package for the analysis.

The predictive performance was evaluated using two different metrics: the accuracy, i.e. the fraction of patients correctly classified, and the F1-score averaged over the two classes (weighted average due to class imbalance), i.e. the weighted average of precision and recall for each class. We performed leave-one-out cross validation to test the models, i.e. employing each patient to validate a model obtained from all other patients. We repeated the analysis on the datasets from both COVID-19 waves, to test the selected clinical variables.

#### Imputation of missing values

We implemented a method for imputation of missing values based on a nearest neighbour approach. For each patient $p_i$, a distance to all other patients is computed. Then each missing clinical value in $p_i$ is completed by copying the corresponding value in the closest $p_j$ that has that clinical variable measured. The distance measure used was cosine similarity^52^ among the two vectors of clinical variables, after removing all variables missing in at least one of the two patients. This distance has the advantage that it does not depend on the number of dimensions of the vector. The data was again standardised before missing value imputation.

#### Clustering

After completing the missing values, we also perform clustering of all clinical variables to provide a better view over the biomarkers selected. This step also provides a means to validate the data collected and the missing value computation. We use agglomerative hierarchical clustering^52^ for this purpose, with the number of clusters fixed at 27. This was chosen empirically since it allowed for four of the six selected clinical variables to belong to separate clusters, without fragmenting too much the variable space. Agglomerative clustering groups the data in a bottom up manner, by joining iteratively the two closest groups, and stopping when that number of clusters is reached. Again we used scikit-learn, with Euclidean distance and Ward linkage.

## Supplementary Information

Supplementary Information.

## Data Availability

The Python implementation of the feature selection algorithm is freely available on GitHub: https://github.com/alinasirbu/clinicalVariableSelection.
