# Supplementary material for: Early outcome detection for COVID-19 patients
Source: Sci Rep. 2021 Sep 16;11:18464. doi: 10.1038/s41598-021-97990-1 (PMC8446000; doi:10.1038/s41598-021-97990-1)
Supplement: Supplementary file 1 — Supplementary Information. [file 41598_2021_97990_MOESM1_ESM.pdf]

Early Outcome Detection for COVID-19 Patients
Supplementary Material

Alina Sîrbu, Greta Barbieri, Francesco Faita, Paolo Ferragina, Luna Gargani, Lorenzo Ghiadoni, Corrado Priami

1 Introduction

This document provides supplementary information for the manuscript with the same title. Section 2 lists the clinical variables contained in our data. Section 4 displays detailed results of the algorithm for feature selection described in the main manuscript. These results formed the basis for selecting six variables to be included in the final models. Section 5 provides further details on the algorithms, discussing its robustness to the parameters set during the experiments. Finally, Section 6 discusses other possible clinical variables that were not considered in the main manuscript.

2 Clinical variables

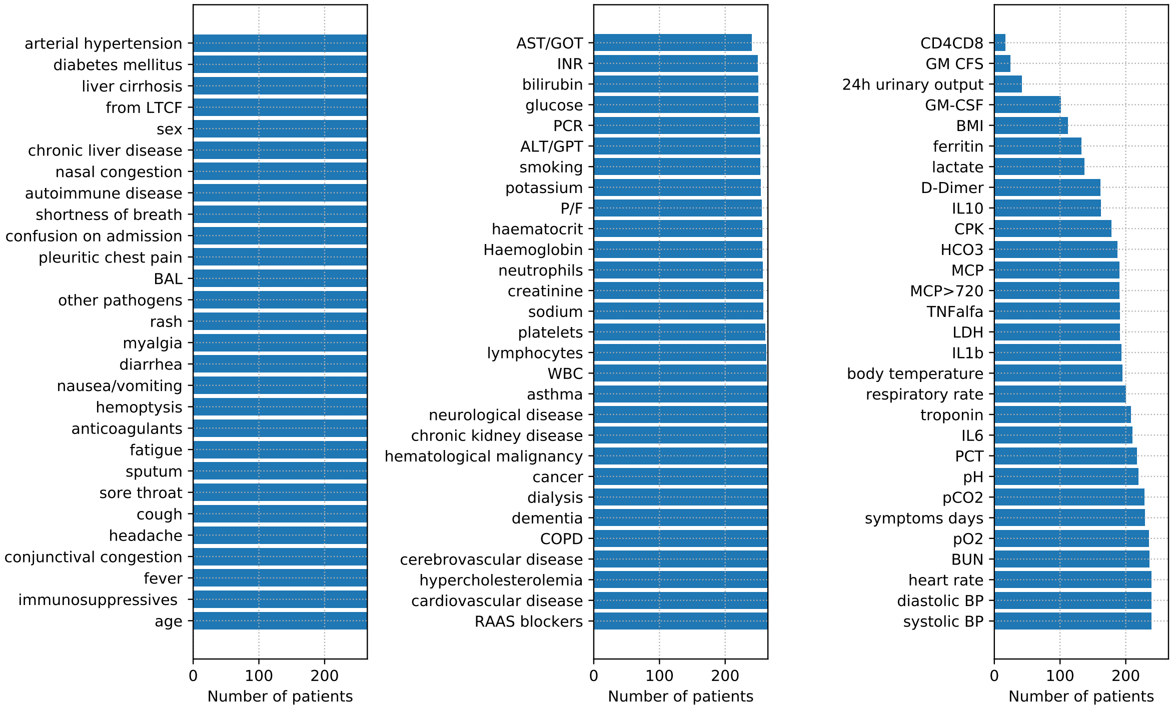

Figure 1: Clinical variables. The bars show the number of patients per each variable in our dataset.

Figure 1 shows the full list of clinical variables in our first wave dataset (overall 313 COVID-19 patients), with bars indicating the number of patients for which each variable is measured. We

can observe that the variables related to preexisting conditions, ongoing therapies and symptoms on admission have a very good coverage. Some laboratory testing variable have patient coverage lower than 50%.

3 Classification with all clinical variables

Before performing feature selection, we have studied the classification performance of five predictive models on all data from the first COVID-19 wave. Since the data contain missing values, a first step was to crop the dataset to obtain a full data matrix. We adopted a method based on coverage: first remove all clinical variables that were available for a fraction of patients lower than a *coverage threshold* , then remove all patients with missing data. We used thresholds of 100%, 90%, 75%, 50%, and the resulting number of patients and features, together with the predictive performance with leave one out cross validation is shown in Table 1. We can observe that the threshold of 90% provides the best performance, with the RF model 86% accuracy, followed by logistic regression and decision trees at 83%. When decreasing the threshold at 75%, the number of available patients decreases a lot. At this threshold, the logistic regression models displays best accuracy. At the 100% threshold, all models display lower prediction abilities, with best results again for the random forest model, very closely followed by logistic regression. For the 50% coverage thresholds, the data becomes extremely scarce.

Among all models, random forests ad logistic regression appear to have provided the best prediction before feature selection. Since random forests take much longer to train (27.68s versus 1.41s for a full leave one out cross validation run), we decided to employ logistic regression in our feature selection method, which requires model training at every fitness evaluation within the genetic algorithm.

Table 1: Classification performance with Leave one Out Cross Validation on the first wave data, using different coverage thresholds for variables, and thus resulting in a different support for each analysis.

Coverage threshold	N patients	N Variables	Model	F1-Score	Accuracy
100%	265	38	LR	0.772	0.777
100%	265	38	DT	0.729	0.740
100%	265	38	RF	**0.773**	**0.781**
100%	265	38	NB	0.531	0.521
100%	265	38	SVM	0.719	0.751
90%	171	59	LR	0.831	0.830
90%	171	59	DT	0.817	0.830
90%	171	59	RF	**0.856**	**0.865**
90%	171	59	NB	0.417	0.415
90%	171	59	SVM	0.780	0.819
75%	74	68	LR	**0.849**	**0.851**
75%	74	68	DT	0.799	0.838
75%	74	68	RF	0.775	0.824
75%	74	68	NB	0.566	0.514
75%	74	68	SVM	0.726	0.811
50%	8	79	LR	0.417	0.500
50%	8	79	DT	0.481	0.625
50%	8	79	RF	0.417	0.500
50%	8	79	NB	0.417	0.500
50%	8	79	SVM	0.481	0.625

4 Selection of clinical variables and independent validation

The filter/wrapper feature selection algorithm introduced in the main manuscript was applied to different patient subsets from the first wave dataset, using the rest of the patients from the same dataset as external validation data. We repeated the analysis 5 times, following a 5-fold cross validation technique, as described in Figure 2, resulting in 5 different patient cohorts used for clinical variable selection. For each cohort, the algorithm returns a ranking of clinical variables, which can be used to filter the variables by selecting only those at the top of the ranking. The second wave dataset was not employed in the feature selection phase.

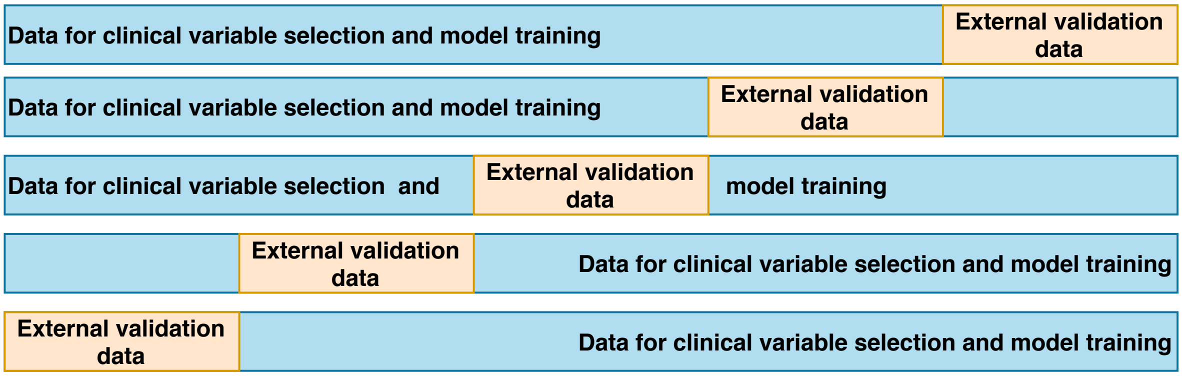

Figure 2: Five-fold cross validation dividing the first wave patients into five folds, and using one fold for external validation and the rest of the folds for clinical variable selection and model training.

Tables 2 to 6 display the top 20 clinical variables for each of the five patient cohorts, together with predictive performance of multiple logistic regression models that employ an increasing number of clinical variables in the ranking order. We show AUC and Accuracy both on training data (the same patients used to generate the ranking, marked in blue in Figure 2) and on external validation data (patients not used to generate the ranking, marked in yellow in Figure 2).

The variables at the top of the raking are very stable: the first two positions are always held by P/F and age, while the third and fourth are shared by COPD, troponin, BUN and myalgia. This is what led us to select these variables for prediction.

The prediction reaches validation AUC values close to 0.8 quite fast at the top of all rankings. If we consider the top 4 variables in each ranking, the average AUC is 0.79 (minimum of 0.77, maximum of 0.83), while when considering 6 variables the average becomes 0.81 (minimum of 0.76 and maximum of 0.84). We finally selected the union of the top 4 variables from each ranking, resulting in 6 total variables, which cover 5 out of the top 6 of each ranking.

We also note that after the initial increase in performance, we typically observe a decrease. In some cases the performance starts to increase again, reaching even perfect classification, but the support (the number of patients) becomes very small, hence we believe these are not relevant.

Table 2: Logistic regression performance with the top 20 clinical variables ranked by the genetic algorithm, on the first cohort. Each row corresponds to a different logistic regression model where all the variables up to the current row are used. For instance, the first row employs only the first clinical parameter, the second row employs the first 2 parameters, and so on. The support column shows the number of patients in the test/validation data, with the deceased count in parenthesis.

Clinical variable	Train AUC	Train Accuracy	Train Support	Validation AUC	Validation Accuracy	Validation Support
P/F	0.69	0.81	203 (49)	0.63	0.73	52 (17)
age	0.77	0.86	203 (49)	0.82	0.87	52 (17)
troponin	0.83	0.89	161 (40)	0.79	0.83	41 (13)
COPD	0.83	0.89	161 (40)	0.83	0.85	41 (13)
creatinine	0.83	0.91	159 (39)	0.80	0.82	40 (12)
BUN	0.84	0.91	145 (34)	0.84	0.87	39 (12)
ferritin	0.90	0.94	84 (18)	0.70	0.70	20 (7)
neurological disease	0.90	0.94	84 (18)	0.78	0.80	20 (7)
myalgia	0.90	0.94	84 (18)	0.74	0.75	20 (7)
respiratory rate	1.00	1.00	69 (12)	0.79	0.83	18 (6)
diastolic BP	1.00	1.00	69 (12)	0.70	0.76	17 (6)
symptoms days	1.00	1.00	62 (10)	0.70	0.76	17 (6)
chronic liver disease	1.00	1.00	62 (10)	0.70	0.76	17 (6)
asthma	1.00	1.00	62 (10)	0.70	0.76	17 (6)
systolic BP	1.00	1.00	62 (10)	0.70	0.76	17 (6)
sodium	1.00	1.00	62 (10)	0.70	0.76	17 (6)
cerebrovascular disease	1.00	1.00	62 (10)	0.70	0.76	17 (6)
autoimmune disease	1.00	1.00	62 (10)	0.70	0.76	17 (6)
BAL	1.00	1.00	62 (10)	0.70	0.76	17 (6)
lymphocytes	1.00	1.00	62 (10)	0.70	0.76	17 (6)

Table 3: Logistic regression performance with the top 20 clinical variables ranked by the genetic algorithm, on the second cohort. Each row corresponds to a different logistic regression model where all the variables up to the current row are used. For instance, the first row employs only the first clinical parameter, the second row employs the first 2 parameters, and so on. The support column shows the number of patients in the test/validation data, with the deceased count in parenthesis.

Clinical variable	Train AUC	Train Accuracy	Train Support	Validation AUC	Validation Accuracy	Validation Support
P/F	0.67	0.79	205 (56)	0.66	0.82	50 (10)
age	0.77	0.84	205 (56)	0.81	0.88	50 (10)
COPD	0.76	0.83	205 (56)	0.88	0.92	50 (10)
BUN	0.82	0.89	183 (45)	0.77	0.86	43 (8)
troponin	0.89	0.93	147 (38)	0.81	0.84	38 (8)
chronic liver disease	0.87	0.93	147 (38)	0.81	0.84	38 (8)
respiratory rate	0.89	0.92	118 (30)	0.86	0.90	31 (5)
diarrhea	0.89	0.92	118 (30)	0.76	0.87	31 (5)
myalgia	0.89	0.92	118 (30)	0.78	0.90	31 (5)
anticoagulants	0.89	0.92	118 (30)	0.76	0.87	31 (5)
PCT	0.89	0.92	104 (29)	0.86	0.90	29 (5)
BAL	0.89	0.92	104 (29)	0.86	0.90	29 (5)
sodium	0.90	0.92	104 (29)	0.86	0.90	29 (5)
ferritin	0.94	0.97	64 (16)	0.69	0.83	18 (2)
diastolic BP	0.94	0.97	63 (16)	0.69	0.83	18 (2)
cerebrovascular disease	0.94	0.97	63 (16)	0.69	0.83	18 (2)
confusion on admission	0.94	0.97	63 (16)	0.69	0.83	18 (2)
D-Dimer	1.00	1.00	33 (7)	1.00	1.00	11 (1)
fever	1.00	1.00	33 (7)	1.00	1.00	11 (1)
symptoms days	1.00	1.00	29 (6)	1.00	1.00	11 (1)

Table 4: Logistic regression performance with the top 20 parameters ranked by the genetic algorithm, on the third cohort. Each row corresponds to a different logistic regression model where all the parameters up to the current row are used. For instance, the first row employs only the first clinical parameter, the second row employs the first 2 parameters, and so on. The support column shows the number of patients in the test/validation data, with the deceased count in parenthesis.

Clinical variable	Train AUC	Train Accuracy	Train Support	Validation AUC	Validation Accuracy	Validation Support
P/F	0.67	0.79	205 (56)	0.66	0.82	50 (10)
age	0.77	0.84	205 (56)	0.76	0.80	50 (10)
COPD	0.80	0.86	205 (56)	0.69	0.80	50 (10)
BUN	0.83	0.90	183 (45)	0.78	0.88	43 (8)
diastolic BP	0.87	0.91	167 (42)	0.78	0.87	38 (8)
myalgia	0.87	0.92	167 (42)	0.76	0.84	38 (8)
respiratory rate	0.85	0.90	140 (34)	0.69	0.82	33 (6)
systolic BP	0.85	0.89	140 (34)	0.69	0.82	33 (6)
troponin	0.91	0.93	119 (29)	0.87	0.89	28 (6)
creatinine	0.91	0.93	118 (29)	0.87	0.89	28 (6)
ferritin	0.90	0.94	68 (13)	0.76	0.83	18 (5)
chronic liver disease	0.90	0.94	68 (13)	0.76	0.83	18 (5)
BAL	0.90	0.94	68 (13)	0.76	0.83	18 (5)
heart rate	1.00	1.00	68 (13)	0.71	0.82	17 (4)
confusion on admission	1.00	1.00	68 (13)	0.71	0.82	17 (4)
platelets	1.00	1.00	68 (13)	0.67	0.76	17 (4)
PCT	1.00	1.00	63 (13)	0.71	0.82	17 (4)
LDH	1.00	1.00	45 (6)	0.75	0.90	10 (2)
potassium	1.00	1.00	44 (6)	0.75	0.90	10 (2)
other pathogens	1.00	1.00	44 (6)	0.69	0.80	10 (2)

Table 5: Logistic regression performance with the top 20 clinical variables ranked by the genetic algorithm, on the fourth cohort. Each row corresponds to a different logistic regression model where all the variables up to the current row are used. For instance, the first row employs only the first clinical parameter, the second row employs the first 2 parameters, and so on. The support column shows the number of patients in the test/validation data, with the deceased count in parenthesis.

Clinical variable	Train AUC	Train Accuracy	Train Support	Validation AUC	Validation Accuracy	Validation Support
P/F	0.68	0.80	204 (52)	0.62	0.75	51 (14)
age	0.79	0.86	204 (52)	0.69	0.80	51 (14)
COPD	0.78	0.86	204 (52)	0.74	0.84	51 (14)
troponin	0.81	0.87	163 (43)	0.80	0.90	39 (10)
respiratory rate	0.85	0.90	135 (33)	0.83	0.89	28 (7)
myalgia	0.85	0.90	135 (33)	0.83	0.89	28 (7)
anticoagulants	0.86	0.90	135 (33)	0.83	0.89	28 (7)
BAL	0.85	0.90	135 (33)	0.76	0.86	28 (7)
systolic BP	0.85	0.89	133 (33)	0.83	0.89	28 (7)
diarrhea	0.86	0.90	133 (33)	0.83	0.89	28 (7)
AST/GOT	0.85	0.90	126 (32)	0.78	0.88	25 (5)
diastolic BP	0.87	0.91	126 (32)	0.78	0.88	25 (5)
BUN	0.87	0.91	117 (29)	0.69	0.81	21 (4)
confusion on admission	0.88	0.92	117 (29)	0.82	0.86	21 (4)
PCT	0.90	0.94	105 (28)	0.80	0.83	18 (4)
neurological disease	0.90	0.93	105 (28)	0.80	0.83	18 (4)
ferritin	0.97	0.99	68 (16)	0.70	0.83	12 (2)
Haemoglobin	0.97	0.99	68 (16)	0.95	0.92	12 (2)
HCO3	1.00	1.00	56 (11)	0.94	0.90	10 (1)
sex	1.00	1.00	56 (11)	0.94	0.90	10 (1)

Table 6: Logistic regression performance with the top 20 clinical variables ranked by the genetic algorithm, on the fifth cohort. Each row corresponds to a different logistic regression model where all the variables up to the current row are used. For instance, the first row employs only the first clinical parameter, the second row employs the first 2 parameters, and so on. The support column shows the number of patients in the test/validation data, with the deceased count in parenthesis.

Clinical variable	Train AUC	Train Accuracy	Train Support	Validation AUC	Validation Accuracy	Validation Support
age	0.70	0.79	212 (56)	0.55	0.68	53 (15)
P/F	0.79	0.86	203 (51)	0.67	0.79	52 (15)
troponin	0.81	0.88	161 (42)	0.77	0.88	41 (11)
myalgia	0.84	0.89	161 (42)	0.77	0.88	41 (11)
COPD	0.83	0.89	161 (42)	0.77	0.88	41 (11)
BUN	0.86	0.92	144 (35)	0.82	0.90	41 (11)
respiratory rate	0.89	0.91	117 (27)	0.92	0.94	32 (8)
symptoms days	0.90	0.93	106 (24)	0.91	0.93	29 (7)
diarrhea	0.91	0.93	106 (24)	0.76	0.86	29 (7)
ferritin	0.95	0.97	62 (13)	0.83	0.94	18 (3)
creatinine	0.92	0.97	62 (13)	0.83	0.94	18 (3)
chronic liver disease	0.96	0.98	62 (13)	0.83	0.94	18 (3)
potassium	0.96	0.98	61 (13)	0.83	0.94	18 (3)
diastolic BP	0.96	0.98	60 (13)	0.60	0.78	18 (3)
neurological disease	0.96	0.98	60 (13)	0.60	0.78	18 (3)
smoking	0.96	0.98	59 (13)	0.46	0.80	15 (2)
chronic kidney disease	0.96	0.98	59 (13)	0.42	0.73	15 (2)
systolic BP	0.96	0.98	59 (13)	0.46	0.80	15 (2)
sex	0.96	0.98	59 (13)	0.42	0.73	15 (2)
shortness of breath	0.96	0.98	59 (13)	0.42	0.73	15 (2)

5 Robustness analysis

The feature selection algorithm obtains the rankings by combining a very large number of solutions resulting from the feature genetic algorithm with different parameters. We opted for this methodology because we observed a good agreement between solutions with different parameters, indicating that the ranking of the clinical variables is robust to the settings of the algorithm. In this section we provide details on this robustness, and discuss the cases where differences can be seen.

The three parameters required by our feature selection algorithm are: N - the number of features selected by each run of the genetic algorithm, p^* - the minimum number of patients for a subset of features and AUC^*, the minimum internal validation AUC under which genetic algorithm solutions are discarded. Tables 7 and 8 compare the rankings obtained when varying these parameters, by showing the position of the top 40 clinical variables in each ranking. Note that $K = 100$ different GA optimisation runs were performed for each column.

The size of the feature set N to be selected by the genetic algorithm has very low impact in the ranking. Larger N values only tend to increase the number of features present in at least one solution, but the top of the rankings remain the same.

The top variables almost always have high ranks. P/F is the most stable, followed by age. Troponin and BUN have high ranks when p^* is small, but as it grows the ranks grow, only to disappear completely for maximum p^*. This is because BUN and Troponin have missing values, and shows that a large threshold on the number of patients filters out the variables with many missing values. Two other clinical variables show this pattern: ferritin and respiratory rate. Ferritin has even more missing values than troponin, hence its usage is difficult in our analysis, while the respiratory rate has quite high ranks in general so it was not considered. A different pattern is seen for some variables that are absent or have low ranks when p^* is small, and then improve their rank as p^* increases. We see this pattern for Creatinine, pre-existing Chronic Liver Disease, or Diastolic Blood Pressure (DBP) for example. This pattern most probably indicates clinical variables that are not very important for prediction when considering all variables, but can compensate for some information lost when clinical variables with missing values are removed. For instance, Creatinine could compensate for the absence of BUN, having fewer missing values. Similarly the DBP could compensate for missing troponin.

No significant differences can be seen at the top of the rankings when moving from a relatively mild AUC threshold of 0.7 to a stricter one of 0.85, with the same most frequent variables.

To further explore the effect of the parameters, Figure 3 shows the value of the AUC computed on internal validation data during the wrapper phase of the algorithm (see Methods section of the main manuscript for details). The boxplots show the distribution over 100 runs of the GA, for each parameter setting, for the first cohort (similar patterns were observed for the other cohorts). We can observe that, in general, larger p^* values result in models with larger AUC, on average. Thus these models are better able to generalise, which was to be expected since the threshold is a limit for the amount of training data available: the more training data, the less overfitting of a specific type of pattern. Furthermore, there is no significant difference between average performance for different values of N, however the distributions appear wider when N is larger.

All in all, the results show some fine differences when zooming into the effect of the algorithm parameters, however each parameter setting brings a contribution and by combining all of them into the final ranking we hope to obtain optimal behaviour.

Table 7: Position of clinical variables in rankings obtained with different algorithm parameters, on cohort 1, with $N = 5$ features selected by the GA.

Clinical variable	Parameter values: p^*, AUC^*											
	50<br>0.7	75<br>0.7	100<br>0.7	125<br>0.7	150<br>0.7	175<br>0.7	50<br>0.85	75<br>0.85	100<br>0.85	125<br>0.85	150<br>0.85	175<br>0.85
P/F	1	1	1	1	1	1	1	1	2	2	1	1
age	8	5	2	2	2	2	15	5	1	1	2	2
troponin	2	2	3	3	21	-	2	2	3	3	8	-
COPD	7	3	6	5	3	5	6	3	6	7	3	4
creatinine	35	19	7	4	4	3	-	-	10	4	10	3
BUN	5	6	4	8	7	-	11	6	4	6	5	-
ferritin	3	4	25	-	-	-	3	4	13	-	-	-
neurological disease	6	13	10	13	11	8	5	8	9	15	4	14
myalgia	36	8	9	9	23	6	-	9	8	12	-	5
respiratory rate	4	7	5	12	-	-	7	7	5	10	-	-
diastolic BP	15	12	13	22	8	-	8	10	-	-	13	-
symptoms days	27	10	12	6	12	-	-	13	18	5	11	-
chronic liver disease	34	-	14	11	5	4	-	-	16	16	6	8
asthma	-	-	-	18	-	14	-	-	-	17	-	13
systolic BP	13	9	8	7	9	-	-	14	11	8	7	-
sodium	-	-	-	-	-	16	-	-	-	-	-	17
cerebrovascular disease	-	14	19	10	10	11	-	17	-	9	17	7
autoimmune disease	-	-	18	-	17	9	-	-	15	-	9	9
BAL	31	-	20	16	20	12	-	-	20	-	-	10
lymphocytes	-	23	24	14	-	7	-	19	12	11	-	16
confusion on admission	10	11	11	20	6	13	12	15	17	-	15	-
hemoptysis	30	-	-	-	-	-	22	-	-	-	-	-
headache	-	-	-	-	-	-	-	-	-	-	-	-
neutrophils	19	-	16	-	-	-	17	-	19	-	-	-
rash	-	-	-	-	26	-	-	-	-	-	-	-
heart rate	16	15	-	15	-	-	9	-	-	20	-	-
PCT	-	-	-	29	22	-	-	-	-	-	-	-
GM-CSF	9	-	-	-	-	-	4	-	-	-	-	-
sputum	-	22	-	-	13	10	-	18	-	-	-	6
ALT/GPT	-	-	-	-	-	-	-	-	-	-	-	-
from LTCF	21	20	26	-	-	25	14	-	14	-	-	18
immunosuppressives	-	-	-	-	-	-	-	-	-	-	-	-
INR	25	-	-	-	24	-	-	-	-	-	-	-
liver cirrhosis	-	-	-	-	-	-	-	-	-	-	-	-
conjunctival congestion	-	-	-	21	-	-	-	-	-	14	-	-
diarrhea	22	-	21	17	18	17	20	-	-	13	-	-
fever	17	18	15	-	27	-	-	12	7	-	14	-
AST/GOT	33	28	-	19	-	-	-	23	-	19	-	-
Haemoglobin	32	-	-	-	-	-	-	-	-	-	-	-
WBC	-	-	-	-	29	23	-	-	-	-	-	-

Table 8: Position of clinical variables in rankings obtained with different meta-parameters, on cohort 1, with $N = 10$ features selected by the GA.

Clinical variable	Parameter values: p^*, AUC^*											
	50 0.7	75 0.7	100 0.7	125 0.7	150 0.7	175 0.7	50 0.85	75 0.85	100 0.85	125 0.85	150 0.85	175 0.85
P/F	2	1	2	1	1	1	3	1	3	1	1	3
age	12	5	1	2	2	2	15	7	2	2	2	2
troponin	6	2	3	4	-	-	8	3	1	3	-	-
COPD	3	3	8	5	3	6	2	2	13	5	5	12
creatinine	14	6	4	3	4	4	18	5	5	4	4	1
BUN	1	7	6	10	29	-	1	9	8	10	29	-
ferritin	5	4	51	-	-	-	4	4	36	-	-	-
neurological disease	7	8	11	8	10	17	7	8	14	13	8	-
myalgia	16	10	5	9	11	7	17	15	6	6	13	16
respiratory rate	4	28	15	45	-	-	6	37	28	37	-	-
diastolic BP	30	21	27	7	7	-	21	26	48	7	6	-
symptoms days	31	12	7	17	39	-	46	18	7	12	35	-
chronic liver disease	62	34	49	14	5	3	54	30	32	16	3	5
asthma	37	13	16	16	6	10	27	11	12	18	7	4
systolic BP	18	32	10	26	13	-	23	28	10	35	21	-
sodium	66	9	-	27	20	21	72	6	-	27	24	8
cerebrovascular disease	34	11	31	11	12	5	57	17	23	11	16	11
autoimmune disease	10	22	14	34	30	8	12	21	19	38	15	15
BAL	23	20	12	6	8	11	40	38	25	8	14	20
lymphocytes	54	19	-	25	38	15	59	12	-	17	-	17
confusion on admission	39	29	37	50	14	9	62	25	31	-	9	7
hemoptysis	32	69	23	22	32	22	24	62	20	25	36	19
headache	24	30	9	36	23	25	16	27	4	30	22	24
neutrophils	15	68	25	23	33	29	10	60	15	31	11	-
rash	42	18	26	43	15	30	31	10	-	24	10	26
heart rate	8	67	46	18	40	-	11	-	24	15	-	-
PCT	50	16	18	60	-	-	37	19	39	47	-	-
GM-CSF	9	-	-	-	-	-	5	-	-	-	-	-
sputum	75	55	47	20	24	12	73	-	26	14	31	23
ALT/GPT	35	17	50	44	60	38	38	20	33	26	33	18
from LTCF	25	14	38	47	53	27	53	13	-	40	-	-
immunosuppressives	63	43	-	19	9	16	69	40	-	32	30	9
INR	20	48	20	33	34	44	35	44	27	52	-	-
liver cirrhosis	48	15	17	39	45	-	36	24	9	49	-	-
conjunctival congestion	46	45	39	12	26	13	49	42	-	9	-	6
diarrhea	13	58	30	-	18	42	13	-	22	-	37	22
fever	55	47	45	46	44	39	60	-	-	29	34	-
AST/GOT	64	57	21	29	49	-	56	52	18	36	-	-
Haemoglobin	76	25	13	32	-	-	74	16	11	48	-	-
WBC	41	23	-	51	22	20	28	14	-	33	32	-

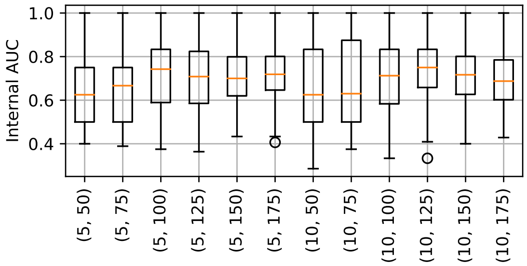

Figure 3: Distribution of internal validation AUC for cohort 1, for different N and p^* values.

6 Exploring other clinical variables

The robustness analysis shows that some clinical variables may be ranked high for some parameter settings, but not appear at the top of the overall ranking. Among these, some also show an increase in the predictive performance in Tables 2 to 6, and are also supported by literature as important parameters: ferritin [1], respiratory rate [2], DBP [3], creatinine [4]. We investigate whether inclusion of these variables in our analysis could have improved prediction. Tables 9 and 10 show an extension of the analysis presented in the main paper to include these variables, for first wave data without and with missing value imputation, respectively. The tables also include the model with 6 parameters only, for comparison reasons. When looking at the original data (Table 9), the parameters respiratory rate and creatinine do not bring any improvement. DBP produces a slight increase in AUC (by 0.004) but with a small decrease in accuracy and with a reduction in the number of patients. Similarly, ferritin produces a slight increase in AUC with stable accuracy, but with a significant decrease in the number of patients. The value of the regression coefficients for these two variables is rather small, indicating a minor contribution. On the imputed data (Table 10), only DBP maintains the slight increase in AUC (again by 0.004) and accuracy remains unchanged. Considering all these factors, we decided not to include any of these variables in our model, as they do not appear to bring any significant improvement in prediction.

Table 9: Logistic regression with leave-one-out cross validation, on the first wave dataset. Each column corresponds to a model where all clinical variables that are assigned a regression coefficient are considered: the first column corresponds to the model using the first six variables (final model from the main manuscript), the second column the first six variables plus diastolic BP, etc.

**Clinical variable**	**Logistic regression coefficients**				
P/F	-1.15	-1.25	-1.04	-1.16	-1.21
age	1.13	1.51	1.63	1.16	1.07
COPD	0.50	0.54	0.48	0.50	0.73
troponin	1.31	1.25	1.20	1.29	0.69
BUN	0.92	0.94	0.85	0.85	0.74
myalgia	-1.21	-1.24	-1.13	-1.20	-1.02
diastolic BP		-0.02			
respiratory rate			0.46		
creatinine				0.12	
ferritin					-0.06
AUC	0.848	0.852	0.846	0.844	0.855
Accuracy	0.903	0.893	0.886	0.897	0.904
Support: total (deceased)	185 (46)	169 (43)	149 (35)	184 (46)	104 (25)

References

[1] Ian Huang, Raymond Pranata, Michael Anthonius Lim, Amaylia Oehadian, and Bachti Alisjahbana. C-reactive protein, procalcitonin, d-dimer, and ferritin in severe coronavirus disease-2019: a meta-analysis. *Therapeutic advances in respiratory disease*, 14:1753466620937175, 2020.

[2] Thomas W Nicholson, Nick P Talbot, Annabel Nickol, Andrew J Chadwick, and Oliver Lawton. Respiratory failure and non-invasive respiratory support during the covid-19 pandemic: an update for re-deployed hospital doctors and primary care physicians. *bmj*, 369, 2020.

[3] Ying-Ying Zheng, Yi-Tong Ma, Jin-Ying Zhang, and Xiang Xie. Covid-19 and the cardiovascular system. *Nature Reviews Cardiology*, 17(5):259–260, 2020.

Table 10: Logistic regression with leave-one-out cross validation, on imputed first wave data. Each column corresponds to a model where all clinical variables that are assigned a regression coefficient are considered: the first column corresponds to the model using the first six variables (final model from the main manuscript), the second column the first six variables plus diastolic BP, etc.

**Clinical variable**	**Logistic regression coefficients**				
P/F	-1.09	-1.09	-1.04	-1.09	-1.08
age	0.91	0.89	0.93	0.90	0.93
COPD	0.55	0.54	0.52	0.55	0.55
troponin	1.45	1.44	1.43	1.46	1.45
BUN	0.74	0.71	0.74	0.76	0.75
myalgia	-0.66	-0.65	-0.64	-0.67	-0.66
diastolic BP		-0.15			
respiratory rate			0.22		
creatinine				-0.02	
ferritin					0.08
AUC	0.817	0.821	0.812	0.817	0.817
Accuracy	0.875	0.875	0.875	0.875	0.875
Support: total (deceased)	265 (71)	265 (71)	265 (71)	265 (71)	265 (71)

[4] Zhaohai Zheng, Fang Peng, Buyun Xu, Jingjing Zhao, Huahua Liu, Jiahao Peng, Qingsong Li, Chongfu Jiang, Yan Zhou, Shuqing Liu, et al. Risk factors of critical & mortal covid-19 cases: A systematic literature review and meta-analysis. *Journal of Infection*, 2020.
